# Influence of platelet-rich plasma (PRP) analogues on healing and clinical outcomes following anterior cruciate ligament (ACL) reconstructive surgery: a systematic review

**DOI:** 10.1007/s00590-021-03198-4

**Published:** 2022-01-12

**Authors:** Jonathon McRobb, Khawaja Hasan Kamil, Imran Ahmed, Fatema Dhaif, Andrew Metcalfe

**Affiliations:** 1 Warwick Medical School, Medical School Building, Coventry, CV4 7HL UK; 2 Warwick Clinical Trials Unit, Coventry, CV4 7AL UK

**Keywords:** Platelet-rich-plasma, Anterior cruciate ligament reconstruction, Biologic augmentation, Systematic review

## Abstract

**Purpose:**

To systematically review the effect of PRP on healing (vascularization, inflammation and ligamentization) and clinical outcomes (pain, knee function and stability) in patients undergoing ACL reconstruction and compare the preparation and application of PRP.

**Methods:**

Independent systematic searches of online databases (Medline, Embase and Web of Science) were conducted following PRISMA guidelines (final search 10th July 2021). Studies were screened against inclusion criteria and risk of bias assessed using Critical appraisal skills programme (CASP) Randomised controlled trial (RCT) checklist. Independent data extraction preceded narrative analysis.

**Results:**

13 RCTs were included. The methods of PRP collection and application were varied. Significant early increases in rate of ligamentization and vascularisation were observed alongside early decreases in inflammation. No significant results were achieved in the later stages of the healing process. Significantly improved pain and knee function was found but no consensus reached.

**Conclusions:**

PRP influences healing through early vascularisation, culminating in higher rates of ligamentization. Long-term effects were not demonstrated suggesting the influence of PRP is limited. No consensus was reached on the impact of PRP on pain, knee stability and resultant knee function, providing avenues for further research. Subsequent investigations could incorporate multiple doses over time, more frequent observation and comparisons of different forms of PRP. The lack of standardisation of PRP collection and application techniques makes comparison difficult. Due to considerable heterogeneity, (*I*^2^ > 50%), a formal meta-analysis was not possible highlighting the need for further high quality RCTs to assess the effectiveness of PRP. The biasing towards young males highlights the need for a more diverse range of participants to make the study more applicable to the general population.

**Trail registration:**

CRD42021242078CRD, 15th March 2021, retrospectively registered.

## Introduction

Anterior cruciate ligament (ACL) rupture is a common injury; 68.6 per 100,000 person years [1]. It often requires surgical intervention and rigorous physiotherapy to allow patients to return to their normal activities. Particularly prevalent in sport, excessive knee rotation can cause ACL rupture, leading to knee pain and instability [2]. The avascular nature of ligamentous tissue and stabilising muscle atrophy result in long recovery times [3]. This inhibits Activities of daily living (ADLs) and can result in prolonged periods of absence from work or /sport.

Platelet-rich plasma (PRP) is a biological augmentation reported to aid healing in damaged cartilage, tendon and ligamentous structures [4]. Resulting from blood separation, PRP consists of autologous mixtures of concentrated platelets and growth factors. Associated with recruitment and proliferation of cells and stimulating angiogenesis [5], early studies have shown improvements in healing and subsequent recovery, indicating use for injuries that require longer recovery periods [6]. Further to this PRP has been reported to reduce pain during recovery in orthopaedic setting [7]. Pain is a major barrier in returning to previous levels of function, either due to effect on mental well-being or ability to complete rehabilitation exercises [8]. In an injury that requires substantial physiotherapy, PRP could improve recovery by facilitating better adherence to physiotherapy protocols, improving knee stability and function.

Much of the literature investigates PRP use in the context of oral and orthopaedic procedures; however, the literature has failed to reach a consensus on its efficacy in ACL reconstruction [9]. This study aims to systematically review the current literature to determine the influence of PRP on the healing and clinical outcomes of ACL reconstructive surgery and compare the methods of PRP collection and application.

## Methodology

**Fig. 1:**
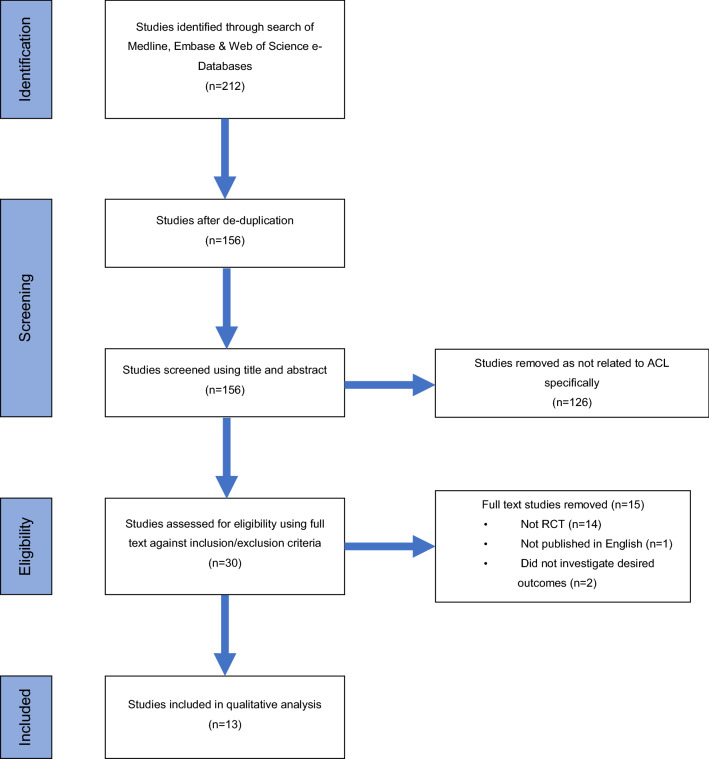
PRISMA flow diagram. Systematic search process and results of the strategy showing number of papers found, removed during screening with reasons and final number of included studies

The review was conducted in accordance with the Preferred reporting items for systematic review and meta-analysis (PRISMA) guidelines. The systematic review was registered on PROSPERO; registration number CRD42021242078. The protocol was designed after consultation with a medical librarian. Studies were then selected based on eligibility criteria (Fig. 1).

### Inclusion

- Randomised controlled trial reporting the effect of PRP on ACL reconstructive surgery outcomes
- Published in English between 2005 and 2021
- Evaluated knee pain, stability, function, vascularisation, inflammation or ligamentization
- Study conducted on humans of any age or gender

### Exclusion

- Reviews and conference abstracts
- Not discussing ACL reconstruction or PRP

### Search strategy

**Fig. 2:**
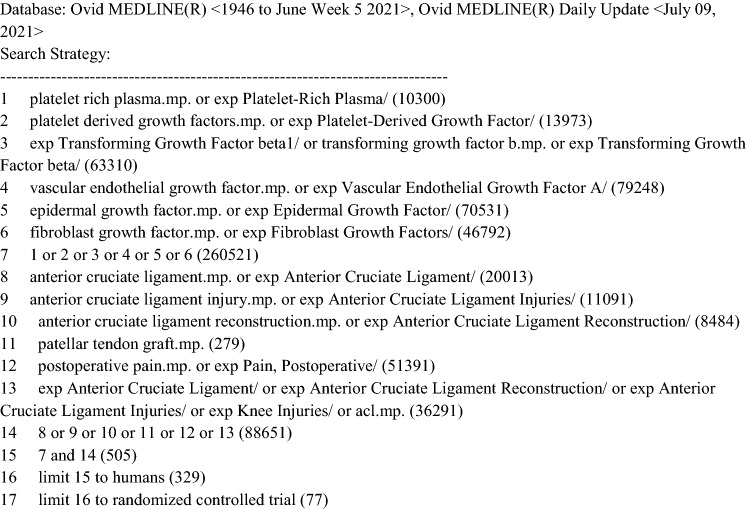
Medline search strategy. All search terms shown along with combinations of terms using “and” or “or” function followed by number of results at each stage

**Fig. 3:**
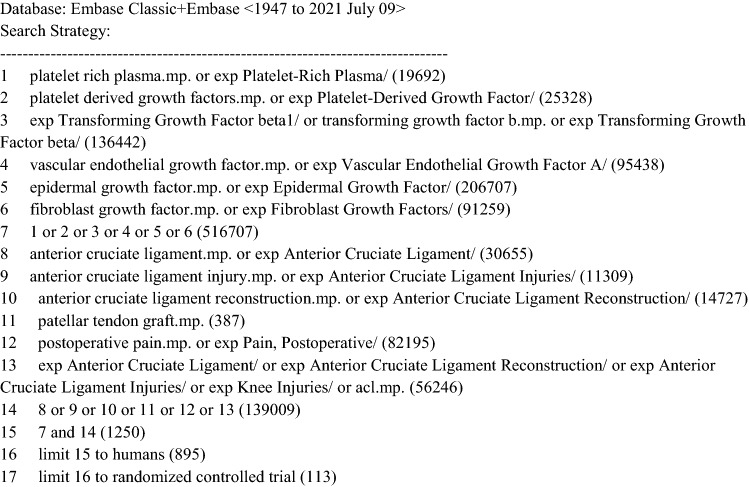
Embase search strategy. All search terms shown along with combinations of terms using “and” or “or” function followed by number of results at each stage

**Fig. 4:**
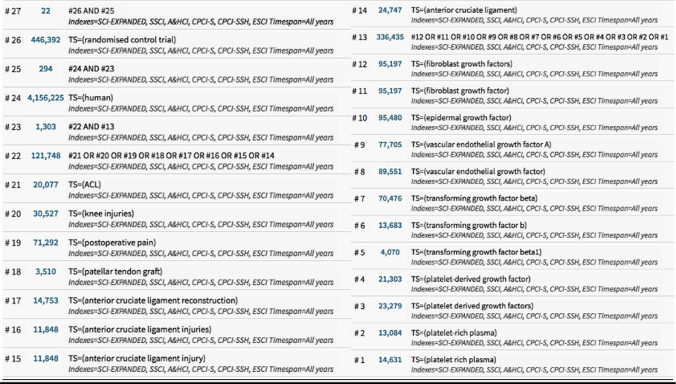
Web of Science search strategy. All search terms shown along with combinations of terms using “and” or “or” function followed by number of results at each stage

A systematic search of Medline, Embase and Web of Science [Figs. 2, 3, 4 respectively] using key words was conducted on 10th of July 2021. Results were exported to Endnote and independently de-duplicated by both authors. Study title and abstracts were then screened against eligibility criteria independently by both authors. Discordance led to inclusion for full text screening to ensure no study was prematurely excluded. Full text screening of the remaining studies was conducted independently by both authors and disagreements remedied by discussion.

### Quality assessment

All included studies were critically appraised by both authors independently using the Critical appraisal skills programme (CASP) RCT checklist [10] with disagreements remedied by discussion. Studies were classed as "good" if answered yes to 9–11 of the CASP criteria and "satisfactory" if answered yes to 6–8 questions.

### Data extraction

Data extraction was conducted in duplicate and inputted into a pilot spreadsheet according to outcomes measured; pain, knee stability, knee function, vascularization, inflammation and ligamentization. The authors, date, year of publication, PRP collection and application method, outcome measured, method of analysis, participant characteristics and results were recorded. Inconsistences were resolved via discussion between authors.

## Analysis

A meta-analysis was considered for pain and knee function outcomes. Given comparable quantitative data, analysis would have involved using a fixed-effects model to calculate Relative risk (RR) and 95% confidence intervals. Following this, a funnel plot and * I*^2^ statistics would have been used to evaluate statistical heterogeneity. Review Manager V.5.3 would have then been utilised to create Forrest plots to summarise meta-analysis results. Where meta-analysis was not possible, a narrative analysis was conducted for each.

## Results

The search identified 212 studies, reduced to 156 after de-duplication. Following title and abstract screening, 126 studies were found not to be investigating ACL reconstruction leaving 30 studies. The full text was reviewed against the inclusion and exclusion criteria resulting in 14 being removed due to not being RCTs, 1 study was not published in English and 2 studies were removed as they did not discuss our outcomes. This left 13 studies and is summarised in Fig. 1. The remaining studies underwent critical appraisal (Table 2). Studies were grouped into overarching healing or clinical themes before export into a data extraction table. Outcomes were grouped as follows: vascularization, inflammation, ligamentization, pain, knee stability and knee function. Methods of PRP collection and application were summarised. A summary of paper characteristics and results is presented in Table 1.

**Table 1:** Summary table of included studies

Title, author and CASP score	Population size (age range) and Platelet-rich plasma method	Outcomes and findings
Autologous platelet-rich plasma gel to reduce donor-site morbidity after patellar tendon graft harvesting for anterior cruciate ligament reconstruction: A randomised, controlled clinical study Cerevellin et al. (2012) 9/11	*N* = 40 (18–29 years old) Two groups: (1) Thrombin activated PRP gel (*n* = 20) (2) Control – No PRP (*n* = 20) Gel applied to patellar and tendon bone harvest site during op	Pain (VAS) pre-op and at 12 months post-op: No significant difference Knee function (VISA-P) scoring pre-op and 12-month post-op: Significantly improved compared to control at 12 months
Patellar tendon healing with platelet-rich plasma: a prospective randomised controlled trial de Almeida et al. (2012) 10/11	*N* = 27 (15–44 years old) Two groups (1) Thrombin activated PRP gel (*n* = 12) (2) Control – No PRP (*n* = 15) Gel applied to patellar tendon harvest site during op	Pain (VAS) immediately post-op: Significantly reduced compared to control (*p* = 0.02) Knee function (IKDC) pre-op and 6 months post-op: No significant difference between the groups (*p* < 0.001)
Efficacy and Tolerability of Progen, a Nutritional Supplement Based on Innovative Plasma Proteins, in ACL Reconstruction: A Multicenter Randomised Controlled Trial Lopez-Vidriero et al. (2019) 9/11	*N* = 68 (18–55 years old) Two groups: (1) Oral supplement containing Porcine PRP (*n* = 34) (2) Control – No supplement (*n* = 34) Supplement provided daily for 90 days	Ligamentization (MRI hypointensity grades 1–4) pre-op and 90 post-op: Significantly higher rates of ligamentization compared to control (*P* = 0.05) Pain (VAS) and Knee function (IKDC) on day 0, 7, 30, 60 and 90 post-op: No significant difference in VAS and significantly improved IKDC at 60 days compared to control (*p* = 0.029)
Value of platelet-rich plasma in osteointegration of tendon graft in anterior cruciate ligament reconstruction Mahdi and Halwas Jhale (2019) 8/11	*N* = 27 (mean age 25.77) Two groups: (1) PRP (*n* = 14) (2) Control – No PRP(*n* = 13) PRP injected into intra-articular graft and femoral tunnel during op	Ligamentization (FIZ signal intensity grades 0–3 using PDW-FatSat and T1w-FatSat-Gad) after 12 weeks post-op: No significant difference in ligamentization Knee function (Lysholm knee scoring scale, Anterior drawer test, Lachmann test and Pivot shift test) at 12 weeks post-op: Significantly improved anterior drawer test (*p* = 0.00), Lachman test (*p* = 0.033) and pivot shift (*p* = 0.014) compared to control. No significance difference in range of movement or lysholm
The impact of platelet-rich plasma on the prevention of tunnel widening in anterior cruciate ligament reconstruction using quadrupled autologous hamstring tendon: A randomised clinical trial Mirzatolooei et al. (2013) 6/11	*N *= 46 (18–40 years old) Two groups: (1) PRP (*n* = 23) (2) Control – No PRP (*n* = 23) Graft submerged in PRP for absorption, remaining PRP injected into bone tunnels during op	Knee stability (KT-1000) pre-op and 3 months post-op: No significant difference
Evaluation of the tibial tunnel after intraoperatively administered platelet-rich plasma gel during anterior cruciate ligament reconstruction using diffusion weighted and dynamic contrast-enhanced MRI Rupreht et al. (2013) 10/11	*N* = 41 (18–50 years old) Two groups (1) Thrombin activated PRP gel (*n* = 21) (2) Control – No PRP (*n* = 20) PRP gel applied to bone tunnels and intra-articular graft during op	Vascularization (GenH) and Inflammation (Apparent Diffusion Coefficient—ADC) at 1-, 2.5- and 6-months post-op: Significant reduction in ADC at 1 month compared to control (*p* = 0.033) and significantly higher Genh at 1 (*p* = 0.019) and 2.5 (*p* = 0.008) months in PRPG group
Magnetic resonance imaging evaluation of patellar tendon graft remodelling after anterior cruciate ligament reconstruction with or without platelet-rich plasma Seijas et al. (2013) 10/11	*N *= 98 (18–65 years old) Two groups: (1) PRP (*n* = 49) (2) Control – No PRP (*n* = 49) Injected in suprapatellar joint during op	Ligamentization (MRI analysis) at 4-, 6- and 12-months post-op: Significantly higher at 4 (*p* = 0.003) and 6 (*p* = 0.0001) months compared to control
Pain in donor site after BTB-ACL reconstruction with PRGF: a randomised trial Seijas et al. (2016) 10/11	*N *= 43 (18–65 years old) Two groups: (1) PRP enriched with growth factors(*n* = 23) (2) Control – No PRP (*n* = 20) Injected into patellar harvest site during op	Pain (VAS) at 1-, 2-, 4-, 6-, 9-, 12- and 24-months post-op: Significant reduction at 1 (*p* < 0.0001) and 2 (*p* = 0.0048) months compared to control
Platelet-rich plasma: Does it help reduce tunnel widening after ACL reconstruction? Vadala et al. (2013) 6/11	*N* = 40 (18–48 years old) Two groups: (1) Thrombin activated PRP gel and PRP with growth factors (*n* = 20) (2) Control – No PRP (*n* = 20) Applied to tunnels during op	Knee function (IKDC, KT-1000) between 10 and 16 months, median 14.7 months post-op: No significant difference
Has Platelet-Rich Plasma Any Role in Anterior Cruciate Ligament Allograft Healing? Valenti Nin et al. (2009) 8/11	*N *= 100 (14–59 years old) Two groups: (1) PRP gel rich in growth factors (*n* = 50) (2) Control – No PRP (*n* = 50) Applied to tibial tunnel and intra-articular graft during op	Ligamentization (MRI signal intensity) 6 months post-op Pain (VAS) 24 h post-op: No significant difference Knee function and stability (KT-1000 and IKDC) at 3-, 6-, 12- and 24-months post-op Inflammation (CRP) at 24 h and 10 days post-op and (PER) pre-op and 24 h post-op: No significant difference
The effect of platelet-derived growth factors on knee stability after anterior cruciate ligament reconstruction: A prospective randomised clinical study Vogrin et al. (2010) 8/11	*N* = 45 (range not given) Two groups: (1) Thrombin activated leukocyte rich PRP gel (*n* = 22) (2) Control – No PRP (*n* = 23) Applied into tunnels and intra-articular graft during op	Knee stability (KT-2000) pre-op, 3- and 6-months post-op: Significant improved at 3 (*p* = 0.035) and 6 (*p* = 0.003) months compared to control
Effects of a platelet gel on early graft revascularization after anterior cruciate ligament reconstruction: A prospective, randomised, double-blind, clinical trial Vogrin et al. (2010) 9/11	*N* = 41 (18–50 years old) Two groups: (1)Thrombin activated PRP gel (*n* = 21) (2) Control – No PRP (*n* = 20) Applied to tunnels and intra-articular graft during op	Vascularization (MRI signal intensity) at 4–6- and 10–12- post-op: Significantly increased 4–6 weeks (*p* < 0.001) at tibial osteoligamentous interface compared to control. No significant difference at intra-articular portion of graft
Effect of Intraoperative Platelet-Rich Plasma Treatment on Post-operative Donor-Site Knee Pain in Patellar Tendon Autograft Anterior Cruciate Ligament Reconstruction: A Double-Blind Randomised Controlled Trial Walters et al. (2018) 7/11	*N* = 44 (range not given, mean age for all 50 patients = 30 ± 12) Two groups (1) Calcium chloride activated PRP gel with patellar bone chips (*n* = 23) (2) Control – No PRP (*n* = 21) Applied to patellar harvest site during op	Pain (VAS) and Knee function (IKDC) at 12 weeks, 6 months, 1 year and 2 years post-op: No significant difference

Included information: Authors, CASP score, titles, type of PRP and region of application, population data, radiological/clinical outcomes with duration of follow-up and main findings

*IKDC*—International knee documentation committee; *PER*—Perimeter; *VAS*—Visual analogue scale; *CRP*—C-Reactive Protein

## Quality assessment

CASP appraisal of methodology involved consideration of blinding protocols, demographical analysis between groups and standardisation of care amongst groups. There were a number of studies displaying deficits in aspects of their methodology. Four studies: Cerevellin et al. [11], Lopez-Vidriero et al. [12], Mirzatolooei et al. [13] and Vogrin et al. [14] did not blind all participants and study personnel to treatment. A further three studies; de Almeida et al. [15], Mahdi and Halwas Jhale [16] and Vadala et al. [17] did not state whether all study personnel and participants were blinded. Four studies: Mahdi and Halwas Jhale [16], Mirzatolooei et al. [13], Vadala et al. [17] and Walters et al. [18] made no reference to demographic analysis between groups thus it was not possible to determine whether the groups were similar at the start of the study. Only Walters et al. [18] did not make reference to post-operative protocols so it is impossible to say whether the groups were treated equally throughout the entire study.

When appraising data from the studies, only three: de Almeida et al. [15], Rupreht et al. [19] and Seijas et al. [20] provided confidence intervals meaning no precision of intervention effect was reported for the remaining studies. Determination of interventional benefit against cost or harms was not possible for eight studies. Mirzatolooei et al. [13], Rupreht et al. [19], Seijas et al. [20], Vogrin et al. [14], Vogrin et al. [21] and Walters et al. [18] did not state adverse outcomes or reference any costs. Vadala et al. [17] and Valenti Nin et al. [22] reported no difference in adverse outcomes between groups but made no references to cost of the intervention and reported no significant difference between groups in any of the outcome measures.

Application of findings to the relevant patient population was scrutinised whereby three studies: Mirzatolooei et al. [13], Vadala et al. [17] and Valenti Nin et al. [22] showed no significant difference between groups for any outcomes at any time, suggesting these interventions did not provide greater value than existing treatments. All studies except Walters et al. [18] had samples that were biased towards young, athletic males. Table 2 provides in depth critical analysis of the included studies using CASP RCT grading. Scores for each individual study are provided in Table 1.

## PRP collection and application methods

Two studies; Vogrin et al. [21] and [14], used the same apparatus for collection. Ten studies isolated platelets via centrifugation (range: 5–15 min and 1500–3200 rpm) whilst de Almeida et al. [15] used filtration separation. Seijas et al. [28] did not state method of separation. Five studies recorded platelet levels within PRP; de Almeida et al. [15] (1,185,166/mm^3^ ± 404,472/mm^3^), Vogrin et al. [14] (average: 962 G/l range: 552–1326), Valenti Nin et al. [22] (average: 837 × 10^3^/mm^3^), Walters et al. [18] (2–3 × above baseline) and M. Mahdi and Halwas Jhale [16] (5.0–7.0 platelets per preparation). Lopez-Vidriero et al. [12] stated the contents of the supplement sachets; 2500 mg of chondroitin sulphate, 300 mg of porcine PRP, 50 mg hyaluronic acid and 40 mg Vitamin C. The remaining studies did not report platelet levels within PRP.

Six studies; Valenti Nin et al. [22], Rupreht et al. [19], Cerevellin et al. [11], de Almeida et al. [15], Vogrin et al. [21] and Mahdi and Halwas Jhale [16] employed methods to maximise PRP retention at application site. The methods used were peritendon and fat pad sutures, suturing PRP into the internal aspect of graft and no use of arthroscopic fluid. Only Lopez-Vidriero et al. [12] trialled multiple applications of intervention, with oral supplementation provided once daily for a 90-day period (Table 3).

## Clinical outcomes

### Pain

Six studies (*n* = 378): Cerevellin,et al. [11], de Almeida, et al. [15], Lopez-Vidriero et al. [12], Seijas et al. [20], Valenti Nin et al. [22] and Walters et al. [18] measured the effect of PRP application on pain during Activities of daily living (ADLs). All studies reported a mean patient Visual analogue scale (VAS) [23] scored out of ten with ten being the highest level of pain. Significance was set at *p* < 0.05 except Lopez-Vidriero et al. [12] who used *p* ≤ 0.05.

Two studies found that PRP caused a significant reduction in pain. De Almeida et al. [15] (*n* = 27) found that PRP significantly reduced pain immediately post-op with 3.8 ± 1.0 (± SD) compared to 5.1 ± 1.4 for the control (*p* = 0.02). Similarly, Seijas et al. [20] (*n* = 43), who measured up to 24 months post-op, observed significantly reduced pain at 1 month with 0.63 compared to 2.58 for the control (*p* < 0.0001) and 2 months with 0.54 compared to 2.21 for the control (*p* = 0.0048). The remaining four studies, of which the maximum period of follow-up was 2 years, reported no significant difference in pain at any interval.

## Knee stability

Five studies (*n* = 258): Mahdi and Halwas Jhale [16], Mirzatolooei et al. [13], Vadala et al. [17], Valenti Nin et al. [22] and Vogrin et al. [14] measured the effect of PRP on knee stability. Mirzatolooei et al. [13], Vadala et al. [17] and Valenti Nin et al. [22] used KT-1000 arthrometers whilst Vogrin et al. [14] used a KT-2000 arthrometer. Mahdi and Halwas Jhale [16] used Lachman’s test [24]. Significance was set at *p* < 0.05.

Two studies found that PRP significantly improved knee stability. Vogrin et al. [14] (*n* = 45), who observed up to 6-months post-op, found significantly improved knee stability at 3-months post-op with a 4.9 ± 1.8 mm displacement compared to 6.1 ± 2.1 mm for the control (*p* = 0.035). This was also found at 6-months post-op with 4.7 ± 1.9 mm compared to 6.7 ± 2.1 for the control (*p* = 0.003). Additionally, M. Mahdi and Halwas Jhale [16] (*n* = 27) found significantly reduced laxity at 12 weeks with 12/14 participants having ≤ 5 mm displacement compared to 6/13 for the control (*p* = 0.033). The remaining three studies, with a maximum follow-up of 2 years, showed no significant improvement in knee stability with the application of PRP.

## Knee function

Seven studies (*n* = 364): Cerevellin et al. [11], de Almeida et al. [15], Lopez-Vidriero et al. [12], Mahdi and Halwas Jhale [16], Vadala et al. [17], Valenti Nin et al. [22] and Walters et al. [18] evaluated the effect of PRP on knee function. All studies analysed knee function via IKDC questionnaires [25], except Mahdi and Halwas Jhale [16], who used Lysholm scores [26] and Cerevellin et al. [11] who used a Patellar tendon Victorian Institute of Sport Assessment questionnaire [27]. Significance was set at *P* < 0.05 for all studies except Lopez-Vidriero et al. [12] who set significance at *P* ≤ 0.05.

Two studies observed significantly improved knee function with PRP use. Lopez-Vidriero et al. [12] (*n* = 68), observing up to 90 days post-op, reported a significant improvement at 60 days with 62.5 ± 11.7 compared to 55.5 ± 11.1 for the control (*P* = 0.029). Further to this, Cerevellin et al. [11] (*n* = 40) measured up to 12 months post-op and found significantly better scores at 12 months with 97.8 ± 2.5 (± SD) compared to 84.5 ± 11.8 for the control (*p* = 0.041). The remaining five studies, with a maximum follow-up of 2 years, found PRP had no significant effect on knee function at any time.

## Parameters of healing

### Vascularization and cellularity

Three studies (*n* = 109); Rupreht et al. [19], Mahdi and Halwas Jhale [16] and Vogrin et al. [21] investigated the effect of PRP application on vascularisation of different parts of the ligament graft. Rupreht et al. [19] investigated the tibial tunnel using a 1.5 T MRI scanner to assess contrast enhancement gradient (G_enh_). Vogrin et al. [21] investigated the tibial osteoligamentous interface and intra-articular graft using contrast-enhanced MRI signal intensity. Mahdi and Halwas Jhale [16] used T1W-FatSat-Gad to assess vascularisation and PDW-Fat-Sat-signal grades for cellularity at the site of osteoligamentous integration (fibrous interzone-FIZ) in the femoral tunnel. Significance was set at *P* < 0.05.

Two studies observed significantly increased levels of vascularisation with PRP administration. Rupreht et al. [19] (*n* = 41) measured up to 6-months post-op. They reported significantly increased vascularization at 1 month with a mean of 2.07 compared to 1.41 for the control (*p* = 0.019) and at 2.5 months, with a mean of 1.64 compared to 1.15 for the control (*p* = 0.008). Vogrin et al. [21] (*n* = 41) measured up to 12-weeks post-op and found significantly increased vascularization of the osteoligamentous interface at weeks 4–6 with 0.33 ± 0.09 vs. 0.16 ± 0.009 for the control (*p* < 0.001).

Mahdi and Halwas Jhale [16] (*n* = 27), who had a minimum follow-up of 12 weeks, found PRP had no significant effect on vascularisation or cellularity at the femoral FIZ.

## Inflammation

Two studies (*n* = 141): Rupreht et al. [19] and Valenti Nin et al. [22] analysed inflammatory parameters after PRP application. Valenti Nin et al. [22] measured C-reactive protein (CRP) and perimeter (PER) of the knee joint (PER1 = patella centre, PER2 = 5 cm above superior patella edge). Rupreht, tal. [19] measured via Apparent diffusion coefficient (ADC), where oedema produced higher values. Significance was set at *P* < 0.05.

Rupreht et al. [19] (*n* = 41) measured up to 6-months post-op and reported significantly reduced ADC values at 1-month post-op with 1.41 (1 $\pm$ 0.1) compared to 1.5 (1 $\pm 0.09)$ for the control (*p* = 0.033).

Valenti Nin et al. [22] (*n* = 100), found no significant difference in CRP up to 10 days post-op and PER 24 h post-op between groups.

## Ligamentization

Three studies (*n* = 266): Lopez-Vidriero et al. [12], Seijas et al. [28] and Valenti Nin et al. [22] investigated the effect of PRP on graft remodelling to determine rate of ligamentization via MRI signal intensity. Significance was set at *P* < 0.05 except for Lopez-Vidriero et al. [12] who used *P* ≤ 0.05.

Two papers found that PRP significantly increased the rate of ligamentization. Seijas et al. [28] (*n* = 98), measuring up to 12-months post-op, showed significantly increased remodelling at 4-months with 39 participants reaching moderately hyperintense as opposed to 23 for the control (*p* = 0.003). This was also seen at 6- months with 46 reaching moderately hyperintense or higher compared to 32 for the control (*p* = 0.0001). Lopez-Vidriero et al. [12] (*n* = 68) measured pre-op and 90 post-op and displayed significantly improved graft maturation at 90 days with 21 participants attaining grade 3 or higher as opposed to 13 for the control (*p* = 0.05).

Valenti Nin et al. [22] (*n* = 100) found no significant difference in ligamentization with PRP application at 6 months post-op.

## Discussion

Success of ACL reconstructive surgery can be measured via clinical features or healing parameters. Literature spanning over a decade has offered insight into whether the use of biological augmentation can enhance these outcomes. These were first described in animal models, where PRP appeared to stimulate the healing processes. In human trials, research into the effects of PRP administration has been conducted in many clinical contexts, with varied results. Following surgery, patients are most concerned with pain levels and knee functionality; hence these outcomes have been widely investigated in the literature. Radiological outcomes provide another measure, allowing for a more rounded and comprehensive analysis of the effect of PRP. This systematic review aimed to collate the clinical and radiological results of RCTs to evaluate whether PRP would benefit those undergoing ACL reconstruction.

Of the clinical outcomes chosen for evaluation: pain, knee stability and function were most widely reported on, encompassing nine of the 13 included RCTs; with radiological considerations in six. Six studies [11, 12, 15, 18, 20, 22] evaluated pain, two of which found PRP reduced VAS scores in the early period post-op [15, 20]. Five studies [13, 14, 16, 17, 22] evaluated knee stability, two of which found PRP reduced anterior–posterior knee laxity [14, 16]. No significant difference was found beyond 6 months. Seven studies [11, 12, 15–18, 22] evaluated knee function, two of which found PRP to improve overall knee function [11, 12]. No significant difference was found beyond 12 months. Three studies [16, 19, 21] evaluated vascularization and cellularity, two of which found PRP to increase rate of vascularization [19, 21]. No significant difference was found beyond 12 weeks. PRP was not found to cause significant change in cellularity levels. Two studies [19, 22] measured the effect of PRP on inflammatory parameters, with only one showing a significant reduction in inflammation [19]. Three studies [12, 22, 28] evaluated ligamentization, two of which found PRP to increase rate of ligamentization [12, 28]. No significant difference was found beyond 6 months.

The inflammatory response following injury or surgery leads to pain, release of inflammatory cytokines and oedema [29]. Pain is subjective, making it a difficult parameter to monitor in a standardised manner. VAS provides some mediation but cannot negate variation in individual pain thresholds. As pain influences ability to carry out rehabilitation protocols [30], it becomes a barrier to full recovery. Reductions in pain would enable improved proprioceptive and strength rehabilitation, therefore potentially increasing the speed and success of recovery. PRP has been shown to reduce pain levels [31]. Of the six studies that measured VAS, de Almeida et al. [15] demonstrated a significant reduction pain at 24 h post-surgery concurring with the results of Seijas et al. [20] at 1- and 2-months post-surgery. Although a number of the remaining studies suggested that PRP reduced pain, this failed to reach statistical significance hence it cannot be conclusively stated whether PRP has a significant effect on pain. As PRP has not been proven to influence pain, further research must be conducted to evaluate any analgesic role it may play.

Method of PRP collection may have an influence on its ability to exert influence on healing and hence clinical parameters. For the method of PRP collection, 10 papers [11, 13, 14, 16–21] used centrifugation whilst de Almeida et al. [15] used filtration. de Almeida et al. [15] produced significant results in VAS whilst 3 of 4 using centrifugation showed no significant change. These different methods may have an effect on the efficacy of PRP however it is difficult to show as there is not enough data on the use of filtration. More studies using filtration should be conducted to ascertain whether this produces any significant change.

Inflammation has been shown to facilitate angiogenesis and production of hyper-vascular granulation tissue during healing [34]. However, inflammatory cytokines and oedema have also been reported to have adverse effects on recovery from ACL reconstruction. It has been shown that inflammatory cytokines can lead to atrophy of the surrounding muscles which adversely effects knee stability and function [32]. Previous studies, such as Anitua et al. [33], have suggested that PRP has an anti-inflammatory effect which could imply useful applications in ACL reconstruction. Of the two studies that measured inflammation, Valenti Nin et al. [22] found that PRP had no effect on CRP or knee swelling whilst Rupreht et al. [19] found significantly reduced knee oedema at 1-month post-op. These results appear contradictory, but this may be due to the different times at which the measurements were taken. Valenti Nin et al. [22] only observed up to 10 days post-op whereas Rupreht et al. [19] measured at 1-, 2.5- and 6-months. This could mean that the anti-inflammatory effects of PRP are delayed and hence were not picked up by Valenti Nin et al. [22].

The lack of significant results beyond one month could be due to PRP only having short-term effects or that the inflammatory phase is receding meaning tangible results will not be seen later in the studies. This is supported by Janssen and Scheffler [35], who state that the proliferative phase is over after 4–12 weeks. Alternatively, these effects could be dependent upon the composition of PRP. Azcarate et al. [36], who furthered the work of Valenti Nin et al. [22], added another intervention group; PRP without leukocytes. This form was found to significantly reduce CRP and swelling suggesting that it had more potent anti-inflammatory effects. This provides another avenue for research, suggesting that composition of PRP could alter its influence over different outcomes. As inflammation plays a key role in vascularisation and healing, the anti-inflammatory effects of PRP could be counterproductive. However, it is excessive inflammation that limits healing and therefore finding the balance between the angiogenic effects and limiting excessive inflammation could be integral for PRP to be beneficial in this area.

Further to this, it has been reported that platelet concentrations should be over 1 × 10^6^/ml [37]; roughly 5 times baseline (whole blood 2 × 10^5^/ml) for PRP to be effective. Five studies [14–16, 18, 22] reported platelet content within PRP concentrations with only de Almeida et al. [15] indicating an average above 1 × 10^6^/ml. This may explain the significant reduction in VAS reported by de Almeida et al. [15] immediately post-op compared to the lack of a significant difference found by Valenti Nin et al. [22] 24 h post-op, who reported an average platelet concentration of 837 × 10^3^/mm^3^. As the remaining seven studies did not record platelet levels, it is impossible to investigate whether their insignificant results could be attributed to using PRP with insufficient platelet concentrations. Further studies should be completed to investigate how platelet concentrations can influence level of clinical benefit within this setting.

Vascularisation is essential for conversion to a functional ligament. In the bone tunnels this aids osteointegration, which roots the ligament providing a stable attachment. In the intra-articular portion, this converts the tendinous structure to resemble the native ACL. Upon grafting, the tendon tissue undergoes remodelling to acquire ligament characteristics, such as higher levels of irregular collagen and proteoglycan bundles that remodel to produce a densely packed, parallel, uniform morphology [38]. Therefore, ligamentization is essential for the graft to achieve the strength and durability required to fulfil the role of the ACL. Hence, the rate of vascularisation and ligamentization have a direct impact on recovery time, improving knee stability and function. This could benefit those looking for a quicker recovery, such as high-level athletes or those who cannot afford time away from work to recover. Studies of the angiogenic effects of PRP have produced positive results, such as those investigating neovascularisation in cardiac muscle [39], suggesting PRP has potential to improve vascularisation in our setting.

Rupreht et al. [19] and Vogrin et al. [21] found no significant increase in intra-articular vascularisation. The lack of significant data could be attributed to a decreased retention of the PRP gel at the intra-articular portion of the graft, limiting its angiogenic influence. Significantly increased levels of bone tunnel vascularisation were observed at 4–6 weeks post-op, with no benefit seen beyond this point. This is supported by the results of Silva and Sampaio [40] who observed no significant difference in vascularisation 3-months post-op. This suggests that PRP is no longer able to influence proceedings. This could be a dose issue as only single applications of PRP are used in our studies with exception of Lopez-Vidriero et al. [12]. Alternatively, much like the effects seen in inflammatory outcomes, this may be due to a transient role of new vasculature. This is supported by Janssen and Scheffler [35] who state that vascularisation occurs in the proliferative phase (4–12 weeks) and once it has played its role it recedes. Therefore, our question becomes not just how much vascularisation is present, but how early it is occurring.

Of the 12 studies that surgically administered PRP, seven [11, 15, 16, 19, 21, 22, 28] described attempts at PRP retention at the site of application. Of these seven studies, all except Valenti Nin et al. [22] reported significant effects of PRP. In comparison, the five studies [13, 14, 17, 18, 20] that did not attempt to retain PRP, three [13, 17, 18] did not report significant effects. This discrepancy may be owed to the time in which PRP is retained at the site, therefore influencing the ability to yield healing benefits. A comparison of effects of PRP with and without retention efforts, could inform on the significance of this factor.

Following vascularisation, ligamentization occurs at 12 weeks and is continuous throughout recovery [41]. Faster ligamentization means that the graft will be able to function as an ACL earlier, decreasing the time taken to transition through the rehabilitation programme. Lopez-Vidriero et al. [12] demonstrated early benefits in ligamentization at 90-days post-op, whilst Seijas et al. [28] demonstrated a more prolonged benefit with significantly higher levels at both 4 and 6 months. This suggests PRP increases the early rate of ligamentization. However, results from Valenti Nin et al. [22] showed no significant difference at the 6-month mark; congruent with later research by Azcarate et al. [36].

Whilst the results of Lopez-Vidriero et al. [12] and Seijas et al. [28] are in agreement, it should be highlighted that the comparison of these results proves difficult due to different forms of PRP being used; Lopez-Vidriero et al. [12] provided oral supplementation of PRP, Seijas et al. [28] using simple injectable PRP and Valenti Nin et al. [22] using PDGF gel. On the other hand, the use of different preparations of PRP may help in determining which form is most suited in improving ligamentization rates. In addition, only Lopez-Vidriero, E., et al. (2019) provided more than one instance of application. This study provided significant improvement in knee function and ligamentization which could suggest repeat applications provides more substantial benefit. Unlike inflammation and vascularisation, ligamentization has been found to continue well beyond 6 months, and the lack of significant data beyond this stage could suggest a dose related issue. Repeat applications of PRP gel should be conducted to determine whether this could augment its influence, or further investigation into the oral supplement should be conducted to determine whether this is a superior form and whether specific preparations of PRP are best suited for this parameter.

The ultimate goal of reconstructing the ACL is to restore stability and function of the knee. However, studies suggest that of those with ACL injuries, only 55% returned to their competitive sport of choice [42]. Success of the surgery depends on a combination of ability to take part in rehabilitation, effected by pain and inflammation, and rate of graft remodelling based on vascularization and ligamentization. Stability is a measure of the ability of the ACL to limit anterior–posterior translocation of the tibia in relation to the femur. Joint laxity has been associated with dysfunctional and injury-prone ACLs [43], hence a stable joint is key to recovery and return to sports. Vogrin [14] showed reduced laxity at 3 and 6 months using KT-2000 arthrometers whilst M. Mahdi and Halwas Jhale [16] also showed significantly reduced laxity at 12 weeks post-op via use of the Lachman’s test. The remaining three studies found no significant difference in stability when measuring using KT-1000 arthrometers. The lack of consistent, statistically significant differences between PRP and control groups means that it cannot be stated that PRP exerts influence over the anterior–posterior laxity of the knee joint. The contrasting results provide an avenue for further research into the influence of PRP on anterior laxity, as an improvement in this outcome could be directly beneficial to those undergoing ACL reconstruction.

On the other hand, knee function provides the yardstick by which all patients will measure the success of their procedure. Thus, demonstration of a tangible difference supplied by PRP is a key outcome. Generally, no significant improvement in IKDC scores was seen when compared to the control. However, Lopez-Vidriero et al. [12] did report a significant improvement compared to control at 60 days post-op, but not at any other date of investigation. In addition, Cerevellin et al. [11] recording significantly improved VISA-P scores at 12 months could indicate some influence on knee function post-op. However, with five of the seven studies showing no statistically significant difference in knee function between groups, this indicates PRP does not have a significant effect on knee function post-op. This being said, it is important to consider that the differing results may be due to the time intervals at which the IKDC questionnaires were completed. Lopez-Vidriero et al. [12] was the only study to record prior to 12 weeks post-op, doing so on three occasions. The significant result of Lopez-Vidriero et al. [12] resides early in the recovery process, indicating that PRP may have an effect, but wears off by later stages. Hence, it would not have been observed at the time intervals used by the other studies. This early influence mirrors the effects demonstrated in vascularisation, inflammation and ligamentization.

Whilst a strong relationship is not established between PRP and improved knee function, there is room for further investigation in this area. Frequency of PRP applications may offer an interesting addition to these experiments, determining whether a single application is limiting its effect. This may explain why the benefits are only seen early in the study and is supported by Tavassoli et al. [44] who showed the efficacy of PRP increased after multiple injections over time. In addition, whilst it may appear that earlier and more regular analysis of knee function would be an improvement for subsequent studies, the restrictive nature of the rehabilitation protocols [45] may limit the amount of knee function that can be evaluated in the earlier stages of healing.

## Limitations of study

The strengths of this study include the use of RCTs, so the data collected are of high quality. The use of three major e-databases ensured much of the applicable literature was found via our search. Two authors independently performed the search and critically appraised the included studies in detail using the CASP criteria limiting individual error.

Comparison between studies proved to be difficult due to the varying methods of PRP application. There appears to be no standardised approach, meaning caution must be taken when comparing results. We suggest a standardised protocol for the collection and application of PRP with assessment of platelet concentration would make inter-study comparison more reliable.

In addition, measurement intervals were inconsistent amongst the studies. As most of PRP’s significant impacts were early on in the healing process, some studies may therefore miss the intervention effect due to prolonged periods between measurement. Using published data on when processes occur, e.g., vascularization, may inform on when data collection should be conducted.

This study aimed to determine PRP effects following ACL reconstruction for application to the wider population. Unfortunately, the literature is heavily biased towards a younger male athletic populous. Whilst it has been established that the incidence of ACL rupture is higher in the athletic population than the general population [46] it has also been shown that females have a 1.5 times greater risk of rupture when compared to male athletes [47]. Therefore, even though the athletic component of the distribution is representative of ACL injury distribution, the lack of female representation makes the results less applicable as a result of potential selection bias. Future research may benefit from incorporating female participants at a higher frequency.

The detailed critical appraisals (Table 2) show further biases of the studies, including the lack of information regarding blinding and demographic data. The specification of “English language” in our inclusion criteria has exposed our study to selection bias. The lack of research into unpublished work and exclusive use of electronic databases will have increased exposure to publication bias.

## Conclusions

PRP appears to exert early influence on healing in the form of vascularisation and granulation tissue formation, culminating in higher rates of ligamentization. Reductions in swelling and CRP levels suggest PRP could be of benefit in the acute stages of recovery. However, long-term effects were not demonstrated suggesting the influence of PRP to be limited. With no consensus reached on the impact of PRP on pain, knee stability and resultant knee function, future research on these areas must be conducted before a conclusion can be made. Future research may benefit from standardising PRP, incorporation of multiple doses, measurement of platelet levels and increased frequency of observation. Alternatively, more comprehensive comparison between different forms of PRP can indicate which is best for each outcome accordingly.

## Data Availability

The datasets used and/or analysed during the current study are available from the corresponding author on reasonable request.

## Appendix

See Figs. 2, 3, 4, Tables 2 and 3.

**Table 2:** CASP critical appraisal of included studies

Author and CASP score	1. Did the trial address a clearly focused research question?	2. Was the assignment of patients to interventions randomised?	3. Were all of the patients who entered the study accounted for at its conclusion?	4. Were patients, health workers and study personnel ‘blind’ to the treatment?	5. Were the study groups similar at the start of the randomised control trial?	6. Apart from the experimental intervention, did each study group receive the same level of care (that is, were they treated equally)?	7. Were the effects of intervention reported comprehensively?	8. Was the precision of the estimate of the intervention or treatment effect reported?	9. Do the benefits of the experimental intervention outweigh the harms and costs?	10. Can the results be applied to your local population/in your context?	11. Would the experimental intervention provide greater value to the people in your care than any of the existing interventions?
Cerevellin et al. (2012) 9/11	Yes: Investigated whether PRP reduced patellar tendon donor-site morbidity in ACL reconstruction	Yes: 40 patients randomised to two groups	Yes: All patients accounted for	No: Post-operative assessment carried out by non-blinded examiners	Yes: No significant difference between groups (age, gender and time between injury and treatment)	Yes: All received same surgical and post-operative protocol	Yes: Power calculated (80%). Pain and knee function measured clearly and presented. No missing data. Biased towards young, athletic males, no female representation. Unpaired student’s t test used. P values stated if significant difference present	No: CI not provided	Yes: No significant difference in adverse outcomes. No mention of cost	Yes: Included patients requiring ACL reconstructive surgery. The age range was 18–29. Only young, male, athletic patients included	Yes: Significant improvement in VISA-P scores
de Almeida et al. (2012) 10/11	Yes: Investigated patellar tendon healing using PRP after ACL reconstruction	Yes: 27 patients randomised to two groups	Yes: All patients accounted for	Can’t tell: No mention of patient blinding	Yes: No significant difference between groups (age, gender and associated lesions)	Yes: All received the same surgical and post-operative protocol	Yes: Power calculated (value not stated). Pain and knee function measured and presented clearly. No missing data. Biased towards young, active males, small female representation. Statistical analysis used student t-test or Mann–Whitney U test. All P vales were provided	Yes: CI provided	Yes: No significant difference in adverse outcomes. No mention of cost	Yes: Included patients requiring ACL reconstructive surgery. The age range was 15–44. 88.9% were active males, 11.1% were active females	Yes: Significant improvements in VAS scores
Lopez-Vidriero et al. (2019) 9/11	Yes: Investigated effects of PRP oral nutritional supplement on healing and clinical outcomes following ACL reconstruction	Yes: 72 patients randomised to two groups	Yes: All patients accounted for	No: Patients, therapists and treating physicians not blinded	Yes: No significant difference between groups (age, BMI, gender, clinical characteristics, affected knee and pre-operative measures)	Yes: All received the same surgical and rehabilitation protocol	Yes: Power calculated (90%). Pain, knee function and ligamentization measured and presented clearly. Results not reported for every follow-up. Biased towards young, athletic males. Unpaired t testing, Fisher exact test, paired-sample t test, variance of repeated and Mann–Whitney U test used. P values stated if significant difference present	No: CI not provided	Yes: No significant difference in adverse outcomes. Considers but no analysis of cost	Yes: Included patients requiring ACL reconstructive surgery. The age range was between 18–55 years old. 83% of the sample were male	Yes: Significant improvement in IKDC scores and measures of ligamentization
Mahdi and Halwas Jhale (2019) 8/11	Yes: Investigated the effects of PRP on ligamentization and knee function	Yes: 27 patients randomised to two groups	Yes: All patients accounted for	Can’t tell: Did not state whether the investigators were blinded	Can’t tell: No demographic analysis was conducted	Yes: All received the same surgical and post-operative protocol	Yes: Power not calculated. Vascularization and knee function measured and presented clearly. No missing data. Biased towards young, active males, no female representation. Chi-squared test and Fisher’s exact test used. P values were reported	No: CI not provided	Yes: Higher instance of adverse effects with PRP administration but clinical benefits observed. No cost information given	Yes: Included patients requiring ACL reconstructive surgery. Mean age was 25.77 years old. Only young active males included	Yes: Significant improvements in anterior drawer test, Lachman test and pivot shift test
Mirzatolooei et al. (2013) 6/11	Yes: Investigated PRP effect on tunnel widening and knee stability after ACL reconstruction	Yes: 50 patients randomised to two groups	Yes: All patients accounted for	No: Not statement regarding blinding	Can’t tell: No demographic analysis was conducted	Yes: All received the same surgical and post-operative protocol	Yes: Power not calculated. Knee stability measured and presented clearly with no missing data. Biased towards young, active males. t-testing and Pearson’s chi squared tests used. P values were reported where significance was reached	No: CI not provided	Can’t tell: No significant difference in benefits reported. No adverse outcomes or costs reported	Yes: Included patients requiring ACL reconstructive surgery. Age range between 18–40 years old. 4 women were included compared to 42 males	No: No significant improvement in stability between groups
Rupreht et al. (2013) 10/11	Yes: Investigated effect of PRP on tibial tunnel remodelling after ACL reconstruction	Yes: 50 patients randomised to two groups	Yes: All patients accounted for	Yes: Patients and radiologist blinded	Yes: No significant difference between groups (gender, age, injury site and BMI)	Yes: All received the same surgical and post-operative protocol	Yes: Power not calculated. Vascularization and inflammation measured and presented clearly with no missing data. Biased towards males. Mann–Whitney testing, Friedman two-was analysis of variance and Wilcoxon’s signed ranks test used. All P values reported	Yes: CI provided	Can’t tell: No reporting of adverse outcomes or costs	Yes: Included patients requiring ACL reconstructive surgery. Age range 18–50 years old with 31 males and 19 females	Yes: Significant improvements in ADC values and average Genh values
Seijas et al. (2013) 10/11	Yes: Investigated effect of PRP on patellar tendon graft remodelling after ACL reconstruction	Yes: 100 patients randomised to two groups	Yes: All patients accounted for	Yes: The patients and radiologist blinded	Yes: No significant difference between groups (age, comorbidities, gender, height and weight)	Yes: All received the same surgical and post-operative protocol	Yes: Power not calculated. Ligamentization measured and presented clearly with no missing data. No demographic data provided. Mann–Whitney U testing used. P values were provided	No: CI not provided	Yes: No significant difference in adverse outcomes. No mention of cost	Yes: Included patients needing ACL reconstructive surgery. The age range was 18–65 years old	Yes: Significant improvements in remodelling
Seija et al. (2016) 10/11	Yes: Investigated effect of PRGF on anterior knee pain at donor site after ACL reconstruction	Yes: 43 patients randomised to two groups	Yes: All patients accounted for	Yes: Patients and study personnel blinded	Yes: No significant difference between groups (age, sex, occupation, education, smoking habits and sporting activity level)	Yes: All received the same surgical and post-operative protocol	Yes: Power calculated (70%). Pain measured and presented clearly with no missing data. Biased towards young active males. Chi-squared statistics and the Hochberg method used. All P values were reported	Yes: CI provided	Can’t tell: No information on costs or harms	Yes: Included patients requiring ACL reconstructive surgery. The age range was 18–65 years old with 37 males and 6 females	Yes: Significant improvement in VAS scores
Vadala et al. (2013) 6/11	Yes: Investigated effect of PRP on femoral and tibial tunnel widening following ACL reconstruction	Yes: 40 patients randomised to two groups	Yes: All patients accounted for	Can’t tell: No mention of patient blinding	Can’t tell: No demographic analysis conducted	Yes: All received the same surgical and post-operative protocol	Yes: Power calculated (90%). Knee function and stability measured and presented clearly with no missing data. Biased towards young athletic males. Chi-squared and the Fisher exact test used. P values were stated if significant difference found	No: CI not provided	Can’t tell: No additional complications and no significant benefit observed. No mention of cost analysis	Yes: Included patients requiring ACL reconstructive surgery. The age range was 18–48 years old (median 34.5 years old). All patients were male	No: No significant improvement in function
Valenti Nin et al. (2009) 8/11	Yes: Investigated effect of PDGF on healing and clinical outcomes post ACL reconstruction	Yes: 100 participants randomised to two groups	Yes: All patients accounted for	Yes: Patients and study personnel blinded	Yes: No significant difference was found between groups (sex and age)	Yes: All received the same surgical and post-operative protocol	Yes: Power not calculated. Inflammation, pain, knee function and stability measured and presented clearly. Some KT-1000, IKDC and MRI analysis data missing. Biased towards young athletic males. Student t testing, Mann–Whitney U test and Kolmogorov test used. P values not stated for IKDC	No: CI not provided	Can’t tell: No differences in associated injuries, no significant benefit observed. No mention of cost analysis	Yes: Included patients requiring ACL reconstructive surgery. Age range 14–59 years old. There were 22 females and 78 males	No: No significant improvement in inflammatory measures (CRP, PER)
Vogrin et al. (2010) 8/11	Yes: Investigated the effect of PRP on knee stability after ACL reconstruction	Yes: 50 patients randomised to two groups	Yes: All patients accounted for	No: The study was unblinded	Yes: No significant difference between groups (sex, age and BMI)	Yes: All received the same surgical and post-operative protocol	Yes: Power not calculated. Knee stability measured and presented clearly. IKDC scores missing. Biased towards young athletic males. Chi-squared test, Mann–Whitney rank test and linear regression method used. All P values were reported	No: CI not provided	Can’t’ tell: No mention of adverse effects or cost	Yes: Included patients requiring ACL reconstructive surgery. Age range 18–50 years old. There were 30 males and 17 females	Yes: Significant improvement in stability
Vogrin et al. (2010) 9/11	Yes: Investigated effect of PRP on vascularisation rates at the osteoligamentous interface and intra-articular graft portion	Yes: 100 patients randomised to two groups	Yes: All patients accounted for	Yes: Patients and study personnel blinded	Yes: No significant difference between groups (sex, age, injury site, BMI and pre-operative platelet count)	Yes: All received the same surgical and post-operative protocol	Yes: Power not calculated. Vascularization measured and presented clearly with no missing data. Biased towards young, athletic males. Chi-squared test and Mann–Whitney rank test used. All P values were reported	No: CI not provided	Can’t tell: No information on costs or harms	Yes: Included patients who required ACL reconstructive surgery. Age range was 18–50 years old with 31 males and 19 females	Yes: Significant improvement in vascularization
Walters et al. (2018) 7/11	Yes: Investigated effect of PRP on healing and clinical parameters post ACL reconstruction	Yes: 50 patients randomised to two groups	Yes: All patients accounted for	Yes: Patients and study personnel blinded	Can’t tell: Demographic data not displayed or analysed	Can’t tell: All received the same surgical protocol. No mention of post-operative protocol	Yes: Power estimated (80%). Pain and knee function measured and presented clearly with no missing data. Biased towards the active females. Mixed-model analysis of variance and t-testing used. All P values were reported	No: CI not provided	Can’t tell: No information on costs or harms	Yes: Included patients requiring ACL reconstruction. Age range not provided	Yes: Significant improvement in kneeing pain and IKDC

Answers to the 11 CASP critical appraisal questions provided with reasoning and final score. Studies were deemed adequate if answered "yes" for nine or more questions and partially adequate if answered "yes" to six to eight questions

**Table 3:** Comparison of PRP collection and administration

Paper	PRP collection method	PRP administration method
Cerevellin et al. (2012)	Apparatus: Gravitational platelet separation system II Separation: Centrifugation 3200 rpm for 15 min Concentration of platelets in PRP: Not stated	PRP form: Gel: Allowed to gel 2–3 min prior to application Location and method of application: Patellar tendon and bone plug harvest site. Stabilised using peritendon suture Volume: 70% of bone gap filling considered sufficient Number of applications: 1
de Almeida et al. (2012)	Apparatus: Haemonetics MCS + 9000 cell separator with platelet apheresis kit (filtration) Separation: Filtrated Concentration of platelets in PRP: 1,185,166/mm^3^ ± 404,472/mm^3^ (WBC: 0.91/mm^3^ ± 0.81)	PRP form: Gel Location and method of application: Patellar tendon harvest site. Sutured into tendon via peritendon suture. Fat pad sutured to prevent gel infiltration into knee joint Volume: 20-40 ml. Harvest site completely filled Number of applications: 1
Rupreht et al. (2013) *Details unclear, collected from studies [48, 49]	Apparatus: Biomet GPS kit (Warsaw, IN) Separation: Centrifugation for 15 min, speed not stated Thrombin collection: 200 rpm for 5 min Concentration of platelets in PRP: Not stated	PRP form: Gel Location and method of application: 1 ml applied to femoral and tibial tunnels and 3 ml onto graft after insertion. Procedure done without arthroscopic fluid to prevent washout Volume: 5 ml Number of applications: 1
Vogrin et al. (2010)	Apparatus: Magellan autologous platelet separator (Medtronic biologic therapeutics and diagnostics, Minneapolis, Minn., USA) Separation: Centrifugation, speed and duration not stated (PRP) Concentration of platelets in PRP: Not stated	PRP form: Gel Location and method of application: Graft covered in 4 ml of gel then positioned. Femoral and tibial tunnels received 1 ml gel injection each after positioning with pressure applied to promote graft and bone infiltration. Remnants applied to intra-articular graft portion. Water flow stopped to prevent gel washout Volume: 6 ml Number of applications: 1
Vogrin et al. (2010)	Apparatus: Magellan autologous platelet separator (Medtronic biologic therapeutics and diagnostics, Minneapolis, Minn., USA) Separation: Centrifugation, speed and duration not stated Concentration of platelets in PRP: Average 962 G/l (range 552–1326)	PRP form: Platelet-leukocyte rich gel Location and method of application: Femoral and tibial tunnels after graft fixation Volume: Not stated Number of applications: 1
Vadala et al. (2013)	Apparatus: PRP Fast Biotech kit (MyCells® PPT- Platelet Preparation tube) Separation: Centrifugation, speed and duration not stated Concentration of platelets in PRP: Not stated	PRP form: Gel and liquid Location and method of application: Tibial and femoral tunnels coated in 5 ml of liquid and gel PRP form prior to graft positioning Volume: 20 ml Number of applications: 1
Valenti Nin et al. (2009)	Apparatus: Not stated Separation: Centrifugation at 3000 rpm for 8 min, PPP and fibrinogen removed. Further 6 min at 1000 rpm Concentration of platelets in PRP: Average 837 × 10^3^/mm^3^	PRP form: Gel, allowed to gel for 15 min prior to use Location and method of application: Gel sutured within the graft interior. Remaining volume introduced to tibial tunnel after graft positioning Volume: 4 ml Number of applications: 1
Walters et al. (2018)	Apparatus: PRP separation kit and centrifuge system (ACL PRP; Arthrex) Separation: Centrifugation for 5 min at 1500 rpm Concentration of platelets in PRP: 2–3 × above baseline	PRP form: Gel Location and method of application: PRP mixed with cancellous bone chips and placed into patellar donor site Volume: 3-5 ml Number of applications: 1
Seijas et al. (2016)	Apparatus: Not stated Separation: Centrifugation for 1800 rpm for 8 min Concentration of platelets in PRP: Not stated	PRP form: Gel, allowed to gel for 15–20 min prior to use Location and method of application: 1 ml applied to patellar bone gap and tibial bone gap. 1 ml injected cranially and caudally in harvest gap, percutaneously post closure Volume: 4 ml Number of applications: 1
Mahdi and Halwas Jhale (2019)	Apparatus: Trima Accel Automated blood collection system Separation: Centrifugation, speed and duration not stated Concentration of platelets in PRP: Not stated (5.0–7.0 × 10^7^ platelets isolated per preparation)	PRP form: Liquid form Location and method of application: Post evacuation of arthroscopic fluid, 3 ml injected into femoral tunnel. Remaining 3 ml injected intra-articularly Volume: 6 ml Number of applications: 1
Mirzatolooei et al. (2013)	Apparatus: Arthrex double syringe system Separation: Centrifugation at 1500 rpm for 5 min Concentration of platelets in PRP: Not stated	PRP form: Liquid Location and method of application: Graft submerged in PRP liquid for 5 min (> 50% absorbed) then positioned. Prior to closure PRP in joint evacuated and injected into femoral (2 ml) and tibial (1.5 ml) tunnels Volume: Unclear Number of applications: 1
Seijas et al. (2013)	Apparatus: PRGF technique BTI systems Victoria, Spain Separation: Separation method not stated Concentration of platelets in PRP: Not stated	PRP form: Liquid Location and method of application: PRP injected percutaneously injected into suprapatellar joint after portal suture. Drainage kept closed for 6 h Volume: 8 ml Number of applications: 1
Lopez-Vidriero et al. (2019)	Apparatus: Progen nutritional supplement sachets Separation and use: N/A, Used as oral supplement Concentrations of platelets in PRP: N/A Sachet contents: 2500 mg of chondroitin sulphate, 300 mg of porcine PRP, 50 mg hyaluronic acid and 40 mg Vitamin C	PRP form: Porcine powdered form composed of hydrolysed collagen, porcine plasma proteins, a complex of hyaluronic acid & chondroitin sulphate and vitamin C Location and method of application: Taken orally as solution Volume: 1 sachet daily Number of applications: 90-day duration

Major components of the collection (apparatus, separation and platelet concentration) and administration (form, location/method of application and volume/number of applications) of PRP
