# Correction: Influence of platelet-rich plasma (PRP) analogues on healing and clinical outcomes following anterior cruciate ligament (ACL) reconstructive surgery: a systematic review

**DOI:** 10.1007/s00590-024-04171-7

**Published:** 2025-02-11

**Authors:** Jonathon McRobb, Khawaja Hasan Kamil, Imran Ahmed, Fatema Dhaif, Andrew Metcalfe

**Affiliations:** 1 Warwick Medical School, Medical School Building, Coventry, CV4 7HL UK; 2 Warwick Clinical Trials Unit, Coventry, CV4 7AL UK

Correction to: European Journal of Orthopaedic Surgery & Traumatology (2022) 33:225–253. 10.1007/s00590-021-03198-4

In the original version of this article, Jonathon McRobb and Khawaja Hasan Kamil should have been denoted as joint first authors.

